# Loss of GLTSCR1 causes congenital heart defects by regulating NPPA transcription

**DOI:** 10.1007/s10456-023-09869-6

**Published:** 2023-02-06

**Authors:** Fengyan Han, Beibei Yang, Yan Chen, Lu Liu, Xiaoqing Cheng, Jiaqi Huang, Ke Zhou, Dandan Zhang, Enping Xu, Maode Lai, Bingjian Lv, Hongqiang Cheng, Honghe Zhang

**Affiliations:** 1 Department of Pathology and Women’s Hospital, Research Unit of Intelligence Classification of Tumor Pathology and Precision Therapy, Chinese Academy of Medical Sciences (2019RU042), Zhejiang University School of Medicine, Hangzhou, 310058 Zhejiang China; 2 Key Laboratory of Aging and Cancer Biology of Zhejiang Province, Hangzhou, China; 3 Department of Pathology and Pathophysiology, School of Basic Medical Sciences, Hangzhou Normal University, Hangzhou, China; 4 Department of Pathology and Pathophysiology and Department of Cardiology at Sir Run Run Shaw Hospital, Zhejiang University School of Medicine, Hangzhou, 310058 China; 5 Center for Stem Cells and Regenerative Medicine, Department of Orthopedic Surgery of the Second Affiliated Hospital, Zhejiang University School of Medicine, Hangzhou, 310058 China; 6 Department of Pathology and Department of Medical Oncology of the Second Affiliated Hospital, Zhejiang University School of Medicine, Hangzhou, 310058 Zhejiang China; 7 Cancer Center, Zhejiang University, Hangzhou, 310058 Zhejiang China; 8 Key Laboratory of Disease Proteomics of Zhejiang Province, Hangzhou, 310058 Zhejiang China; 9 Department of Pharmacology, China Pharmaceutical University, Nanjing, 210009 China; 10 Department of Pathology and Women’s Hospital, Zhejiang University School of Medicine, Hangzhou, 310058 Zhejiang China

**Keywords:** CHD, GLTSCR1, Embryonic development, NPPA

## Abstract

Precise and specific spatiotemporal domains of gene expression regulation are critical for embryonic development. Recent studies have identified GLTSCR1 as a gene transcriptional elongation regulator in cancer research. However, the function of GLTSCR1, especially in embryonic development, remains poorly understood. Here, we found that GLTSCR1 was essential for cardiac development because Gltscr1 knockout (Gltscr1^−/−^) led to embryonic lethality in mice with severe congenital heart defects (CHDs). Ventricular septal defect and double outflow right ventricular were also observed in neural crest cells with conditional deletion of Gltscr1, which were associated with neonatal lethality in mice. Mechanistically, GLTSCR1 deletion promoted NPPA expression by coordinating the CHD risk G allele of rs56153133 in the NPPA enhancer and releasing the transcription factor ZNF740-binding site on the NPPA promoter. These findings demonstrated that GLTSCR1 acts as a candidate CHD-related gene.

**Supplementary Information:**

The online version contains supplementary material available at 10.1007/s10456-023-09869-6.

## Introduction

GLTSCR1 locates in chromosome 19q13 in which allelic loss is a frequent event in human diffuse gliomas [1]. So far, most studies focused on the roles of GLTSCR1 in tumorigenesis. For example, GLTSCR1 polymorphisms were associated with the aggressiveness of lung cancer and the progression of oligodendroglioma [2], and the high expression of GLTSCR1 predicted a poor prognosis for prostate cancer [3]. Our previous studies also showed that GLTSCR1 inhibited colorectal cancer metastasis through interacting with BRD4 to control transcription elongation [4, 5]. Recently, GLTSCR1 was reported to mediate the formation of the ncBAF complex [6–8]. BAF complexes are involved in lineage and differentiation in embryonic development by dynamically coordinating specific subunits at different stages [9]. In particular, cardiac-enriched BAF60C complex is required for heart development [10, 11] and knockout of GLTSCR1 like (GLTSCR1 L) led to embryonic lethality in mice [12]. However, the potential functions of GLTSCR1 in cardiac development and congenital heart defects (CHD) are still unknown.

Embryonic development is precise, orderly, and multi-dimensional gene regulation process which is involved in cell proliferation, differentiation, and migration. The deciphering the mechanisms of developmental disorders program (DMDD) [13] showed that nearly 25–30% of gene knockout resulted in intrauterine death in embryo and a series of developmental anomalies were highly correlated with the phenotype of heart development, nervous system, and vascular system development [14]. The mammalian heart is highly finely and specially organized. The confluence between the posterior brain original cardiac neural crest cells (CNCCs) are essential for the formation of outflow tract reconstruction, diaphragm, and valve [15]. However, the dysregulation of development related genes could result in the serious birth defects, especially CHDs are the leading cause of neonatal mortality. Currently, about 400 genes abnormity have been implicated in CHD including cell signaling regulators, transcription factors, and structural proteins that are important for heart development. However, approximately 60% of CHD cases are still unexplained [16], in which the underlying molecular mechanism need be further clarified. Nevertheless, the potential functions of GLTSCR1 as a transcriptional regulator in cardiac development and CHD are still in the blank.

As a member of natriuretic peptide family, NPPA encodes atrial natriuretic peptide (ANP) that dynamically expressing in the chambers of the developing heart with the brain natriuretic peptide (BNP). However, the double mutation of ANP and BNP usually leads to heart morphogenesis defects and pericardial edema [17]. Therefore, NPPA has been considered as an important diagnostic and prognostic biomarker for cardiovascular diseases and congenital malformations [18–20]. So far, multiple regulatory elements of NPPA had been identified, including transcription factor-binding sites [21] and super-enhancer cluster [22]. Otherwise, deletion of transcription factor Zac1 in mice to dysregulate Nppa led to morphological defects including atrial and ventricular septal defect and thin ventricular wall [23]. These studies suggest that highly specific regulation of NPPA is crucial for heart development, while the underlying mechanism of NPPA dynamical expression regulation is poorly understood.

Here, we demonstrated that GLTSCR1 is also essential for heart development by coregulating the expression of NPPA with a CHD risk allele of rs56153133. While loss of GLTSCR1 causes VSD and DORV cardiac defects.

## Results

### Gltscr1 deficiency causes embryonic lethality and inhibits growth

**Fig. 1:**
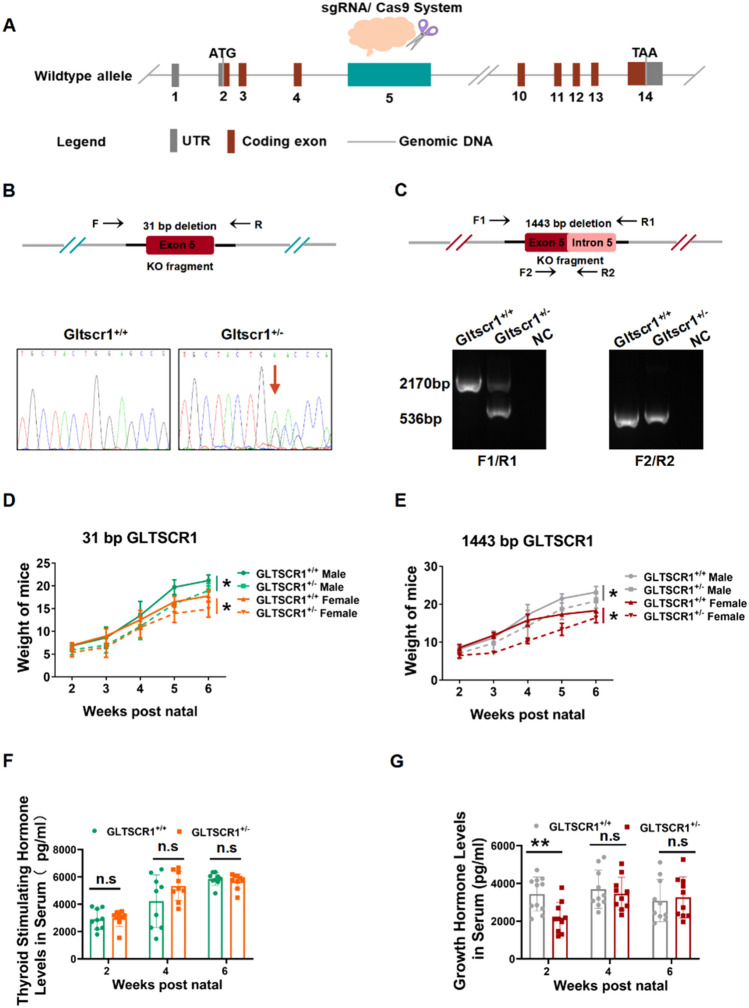
Gltscr1 deficiency causes embryonic lethality and inhibits growth. **A** Schematic overview of Gltscr1 deletion mice model construction. **B** Genotyping of Gltscr1 31 base pairs deletion mice strain. (Top) The schematic diagram of genotyping. (Bottom) Picture shows the Sanger sequence result of 31-bp deletion. The arrow indicates the deletion site. **C** Genotyping of Gltscr1 1443 base pairs deletion mice strain. (Top) The schematic diagram of genotyping. (Bottom) Picture shows the PCR agarose electrophoresis result of 1443-bp deletion. **D**, **E** Growth curve of Gltscr1^+/+^ and Gltscr1^+/−^ in 31-bp (**D**) and 1443-bp (**E**) deletion mouse strains from 2- to 6-week postnatal; mice were separated into male and female. **F**, **G** Thyroid-stimulating hormone (**F**) and growth hormone levels (**G**) in serum at 2-, 4-, and 6-week postnatal were detected by enzyme-linked immunosorbent assay in the 31-bp deletion Gltscr1 mouse strain. *P < 0.05, **P < 0.01 compared to control (two-tailed Student’s t test were used)

To investigate the role of Gltscr1 in embryonic development, we produced two separate Gltscr1 knockout mice strains using CRISPR/Cas9 system. The mouse Gltscr1 gene was located on chromosome 7 with 14 exons, and Gltscr1 knockout mice strain was deleted 31 bases or 1443 bases in the fifth exon separately (Fig. 1A). Genotyping of mice was determined by PCR (Fig. 1B, C). The Gltscr1 heterozygous (Gltscr1^+/−^) male mice were crossed with Gltscr1^+/−^ female mice to generate Gltscr1 homozygous knockout mouse (31 bp deleted Gltscr1^−/−^ or 1443 bp deleted Gltscr1^−/−^). But no Gltscr1^−/−^ strain was obtained, which suggested that homozygous Gltscr1 deletion caused embryonic lethality. Additionally, a smaller body size and weight were observed in Gltscr1^+/−^ mice than in Gltscr1^+/+^ mice (Fig. 1D, E). Next, we assessed the thyroid-stimulating hormone and growth hormone levels in serum at different growth time points postnatal. As shown in Fig. 1F and G, only the growth hormone was significantly lower in the Gltscr1^+/−^ group within two weeks after birth, but it recovered to the same level as the Gltscr1^+/+^ control two weeks later. These data suggest that loss of Gltscr1 results in embryonic lethality and Gltscr1 heterozygosity defect causes growth retardant in early postnatal period.

### Gltscr1-deficient embryo exhibits cardiac defects

**Fig. 2:**
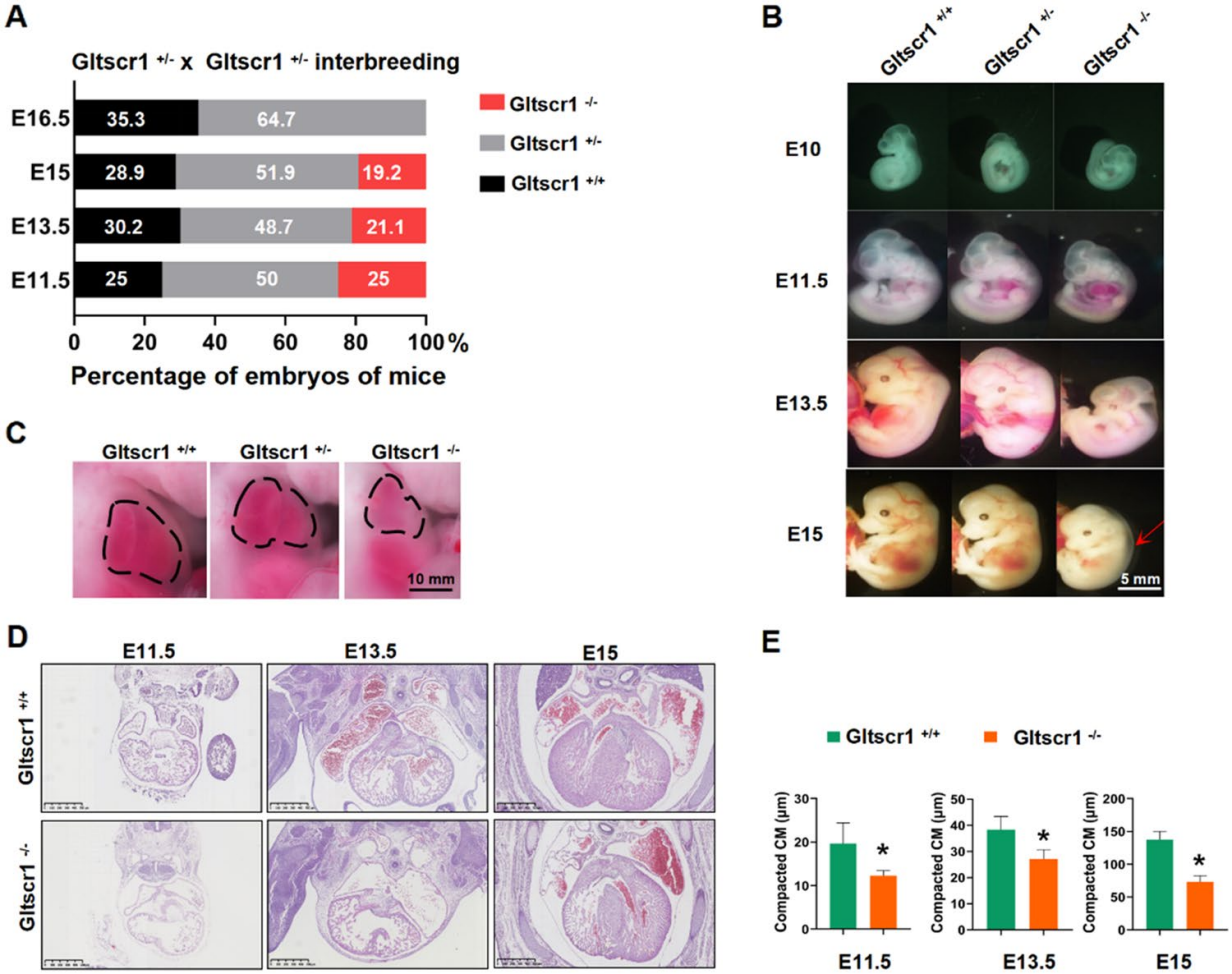
Gltscr1-deficient embryo results in cardiac defects. **A** Genotyping analysis from Gltscr1^+/−^ and Gltscr1^+/−^ interbreeding. **B** Representative images of gross appearance in Gltscr1^+/+^, Gltscr1^+/−^, and Gltscr1^−/−^ embryos at E10 to E15. The arrow indicates the peripheral edema in Gltscr1^−/−^. Scale bar: 5 mm. **C** Representative images of gross appearance in Gltscr1^+/+^, Gltscr1^+/−^, and Gltscr1^−/−^ embryos at E13.5. The dashed area indicates the heart area. Scale bar: 10 mm. **D** Representative H&E staining images of embryonic heart tissues collected at E11.5, E13.5, and E15 from Gltscr1^+/+^ and Gltscr1^−/−^ embryos. Scale bar: 500 µm. **E** Quantification of compacted cardiomyocyte (CM) (**D**) was performed using ImageJ software. Data were shown as the mean ± S.D.; *n* = 18 sections from 3 independent experiments. *P < 0.05 compared to control (two-tailed Student’s t test were used)

The study of deciphering the mechanisms of developmental disorders (DMDD; https://dmdd.org.uk) characterized embryonic-lethal phenotype with a significant statistical correlation with abnormalities in the heart, brain, and vascular system [14]. To elucidate the underlying mechanism of Gltscr1 deficient in embryonic lethality, embryos of Gltscr1^+/−^ at embryonic data 11.5–16.5 (E11.5–E16.5) were collected. Genotyping result showed that the number of Gltscr1^−/−^ at E15 was less than expected Mendelian ration (Figs. 2A and S1A, 19.2% vs. 25%, *P* < 0.001) and no Gltscr1^−/−^ embryo was detected at E16.5. We found that the Gltscr1^−/−^ embryos were smaller than Gltscr1^+/−^ and Gltscr1^+/+^, which presented the development retardant and peripheral edema (Fig. 2B). Otherwise, it is also an indicator of heart failure that embryonic lethality occurs during mid- and late gestation with peripheral edema [24]. In addition, the heart of Gltscr1^−/−^ embryo was smaller with an abnormal impulse (Fig. 2C). Therefore, we further evaluated the histological changes in hearts, which showed that Gltscr1^−/−^ embryos displayed severe cardiac developmental defects, including a thinned ventricular wall with increased trabecular area, ventricular septal defect (VSD), and double outlet right ventricle (DORV) (Figs. 2D, E and S1B).

To demonstrate whether the thinned ventricular wall of Gltscr1^−/−^ embryo was caused by reducing cell proliferation and/or increasing apoptosis, we used immunofluorescence assay to detect ki67 for cell proliferation and Terminal dUTP Nicked-End Labeling (TUNEL) assay to detect apoptosis. There was no significant difference in both ki67 and TUNEL staining between Gltscr1^−/−^ and Gltscr1^+/+^ mice at E11.5, E13.5, and E15 (Fig. S1C, D). Collectively, Gltscr1 deletion caused the cardiac defects and mid- and late gestation embryonic lethality, but not affecting proliferation and apoptosis of embryonic myocardial cells.

### Conditional deletion of Gltscr1 in myocardial cells and cardiovascular endothelial cells

**Fig. 3:**
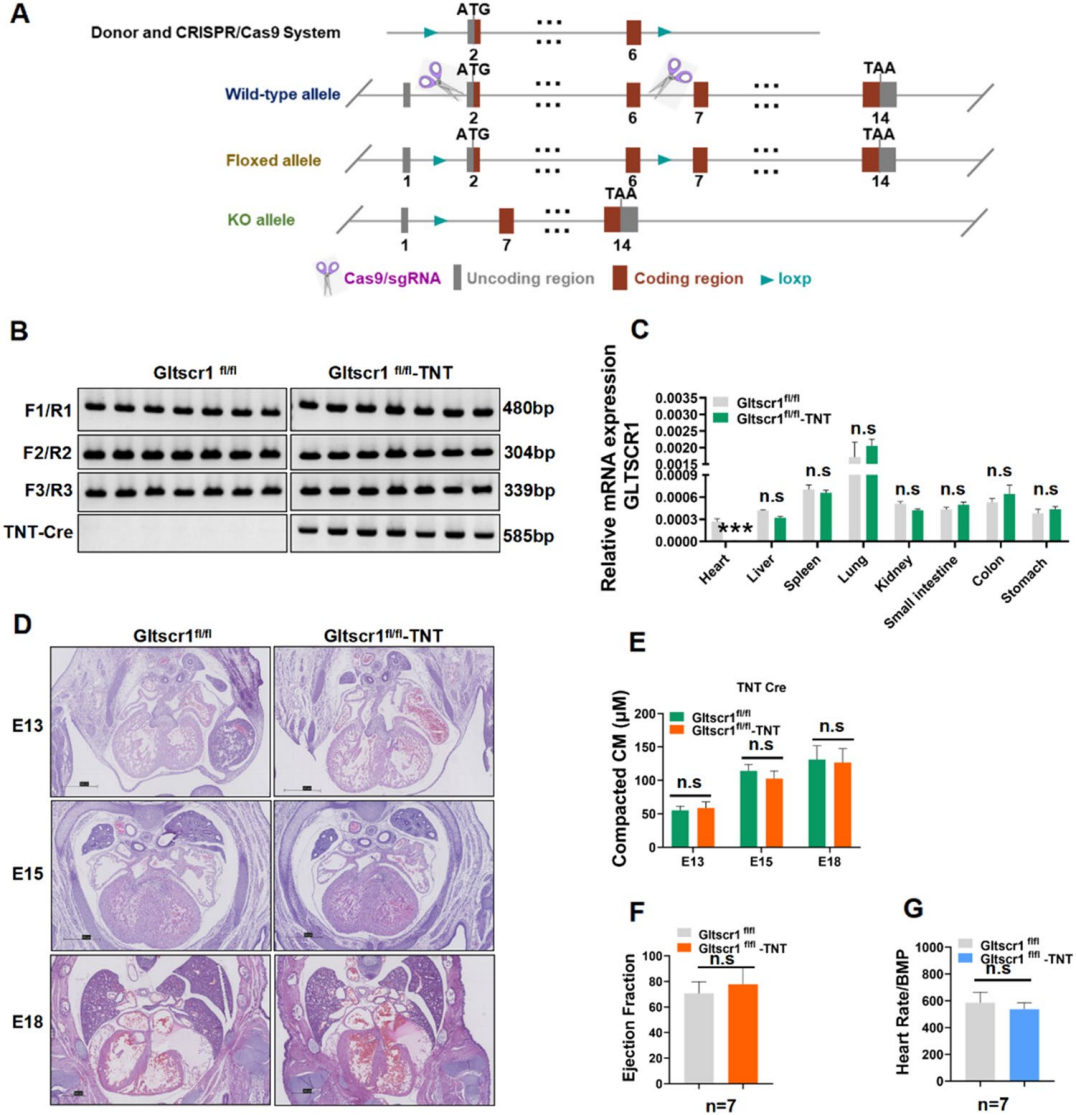
Conditional deletion of Gltscr1 in myocardial cells and cardiovascular endothelial cells. **A** Schematic overview of Gltscr1 conditional deletion mouse model construction. **B** Genotype of conditional deletion Gltscr1 in myocardial cells. **C** RT–qPCR detected the expression of Gltscr1 in Gltscr1^fl/fl^ and Gltscr1^fl/fl^-TNT Cre tissues collected from adult mice. **D** Representative H&E staining images of embryonic heart tissues collected at E13, E15, and E18 from Gltscr1^fl/fl^ and Gltscr1^fl/fl^-TNT Cre embryos. Scale bar: 500 µm. **E** Quantification of (**D**) was performed using ImageJ software. Data were shown as the mean ± S.D.; *n* = 18 sections from 3 independent experiments. Not significant (n.s.) compared to the control (two-tailed Student’s t test was used). **F**, **G** Ultrasonic cardiogram of Gltscr1^fl/fl^ and Gltscr1^fl/fl^-TNT Cre mice. The ejection fraction (**F**) and heart rate (**G**) in myocardial cell conditional Gltscr1 deletion mouse lines, *n* = 7 (two-tailed Student’s t test was used)

To investigate the role of Gltscr1 in cardiac development, we first generated Gltscr1 conditional knockout mouse that was crossed with cardiac muscle-specific expressing TNT Cre mice [25] and endothelial cell-specific expressing CDH5 Cre [26] (Figs. 3A and S2A). After identifying the genotype (Figs. 3B and S2B), we also detected the expression of Gltscr1 in different organs, which showed that Gltscr1 was specifically deleted in heart (Fig. 3C). However, histological analysis of embryos showed the specific Gltscr1 deletion by TNT Cre (Gltscr1^fl/fl^-TNT Cre) and CDH5 Cre (Gltscr1^fl/fl^-CDH5 Cre) did not result in any embryonic lethality or any phenotypic cardiac defect (Fig. 3D, E). Meanwhile, we measured the cardiac function of Gltscr1^fl/fl^-TNT Cre and Gltscr1^fl/fl^-CDH5 Cre mice by ultrasonic cardiogram. Consistent with the histological analysis of embryos, there was no difference in ejection fraction or heart rate in either conditional Gltscr1 deletion mouse line (Figs. 3F, G, and S2C–F). Collectively, these results demonstrated that deletion of Gltscr1 in myocardial cells and cardiovascular endothelial cells were not responsible for mid- and late gestation embryonic development defect in Gltscr1^−/−^ mice.

### Specific deletion of Gltscr1 in neural crest cells cause cardiac defects and neonatal lethality

**Fig. 4:**
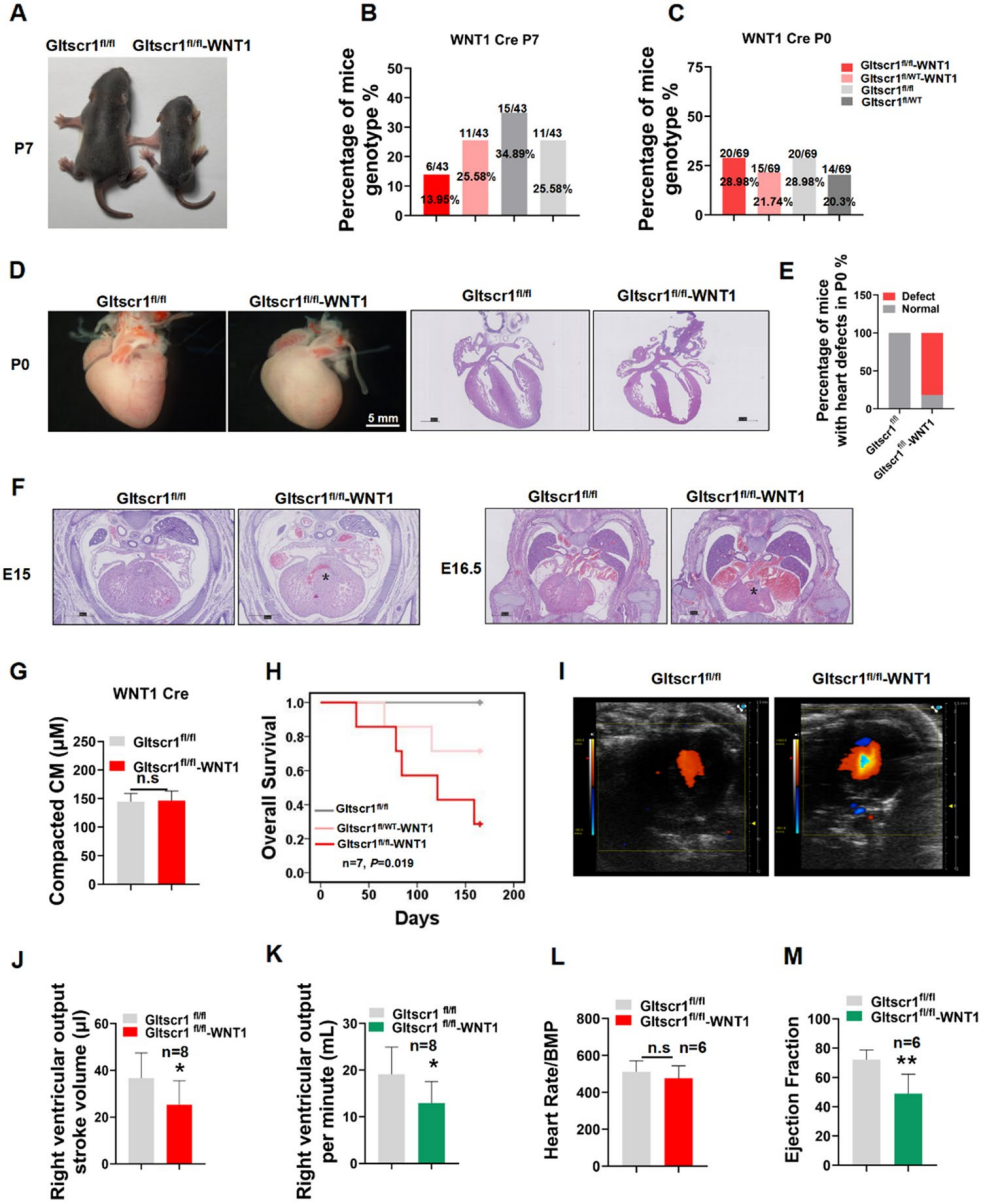
Specific deletion of Gltscr1 in neural crest cells. **A** Representative image of gross appearance in Gltscr1^fl/fl^ and Gltscr1^fl/fl^-WNT1 Cre mice at P7. **B** Genotyping analysis from Gltscr1^fl/wt^ and Gltscr1^fl/wt^-WNT1 Cre interbreeding at P7. **C** Genotyping analysis from Gltscr1^fl/wt^ and Gltscr1^fl/wt^-WNT1 Cre interbreeding at P0. **D** Representative images of gross appearance in Gltscr1^fl/fl^ and Gltscr1^fl/fl^-WNT1 Cre hearts at P0, scale bar: 5 mm. The right pictures are representative H&E staining images of embryonic heart tissues collected at P0. Scale bar: 500 µm. **E** Quantification of the heart defect development percentage of mice at P0, *n* = 11. **F** Representative H&E staining images of Gltscr1^fl/fl^ and Gltscr1^fl/fl^-WNT1 Cre embryonic heart tissues collected at E15 and E16.5, the * at E15 indicates the VSD and the * at E15 indicates DORV. Scale bar: 500 µm. **G** Quantification of (**F**) was performed using ImageJ software. Data are shown as the mean ± S.D.; *n* = 18 sections from 3 independent experiments. n.s compared to control (two-tailed Student’s *t* test were used). Scale bar: 500 µm. **H** Survival curve of Gltscr1^fl/fl^, Gltscr1^fl/wt^-WNT1 Cre and Gltscr1^fl/fl^-WNT1 Cre at 150 days, *n* = 7. **I–M** Ultrasonic cardiogram of Gltscr1^fl/fl^ and Gltscr1^fl/fl^-WNT1 Cre mice. (**I**) Color flow imaging of Gltscr1^fl/fl^ and Gltscr1^fl/fl^-WNT1 Cre mice. Qualification of right ventricular output stroke volume (**J**) and the right ventricular output per minute (**K**) of Gltscr1^fl/fl^ and Gltscr1^fl/fl^-WNT1 Cre mice, *n* = 8. The heart rate (**L**) and the ejection fraction (**M**) in myocardial cell conditional Gltscr1 deletion mouse lines, *n* = 6 (two-tailed Student’s t test was used). *P < 0.05 and **P < 0.01 compared to control (two-tailed Student’s t test were used)

To investigate whether the VSD and DORV cardiac developmental defects in Gltscr1^−/−^ mice were caused by the gene dysregulation of cardiac progenitor cells, we generated a neural crest cell-specific deletion of Gltscr1 by crossing Gltscr1^fl/fl^ mice with WNT1 Cre mice (Fig. S3A), because WNT1 Cre has a recombinase activity both in cardiac and cranial neural crest, specifically expressing in the branchial arches and cardiac outflow tract. Although Gltscr1^fl/fl^-WNT1 Cre embryo could still survive to birth, the newborn Gltscr1^fl/fl^-WNT1 Cre mice at P7 was significantly smaller than control (Gltscr1^fl/fl^) (Fig. 4A). Interestingly, the number of Gltscr1^fl/fl^-WNT1 Cre mice at P7 was less than the expected Mendelian ratio (Fig. 4B, 13.95% vs. 25%, *P* < 0.001). To assess whether the lethality of Gltscr1^fl/fl^-WNT1 Cre mice occurred after birth, we collected the neonatal mice at P0 from Gltscr1^fl/wt^-WNT1 Cre mice crossed with Gltscr1^fl/fl^ mice. As shown in Fig. 4C, the ration of each genotype for neonatal mice conformed to Mendelian ratios (25%). Although no embryonic lethality happened at P0, we observed the abnormal morphology of heart from Gltscr1^fl/fl^-WNT1 Cre mice (Fig. 4D). In parallel, we measured the percentage of mice with heart defects at P0 by histological staining. The result showed that 81.8% of neonatal mice at P0 presented cardiac development defects, which was consistent with the ratio of neonatal lethality at P7 (Fig. 4E). The histological staining result showed that VSD and DORV in Gltscr1^fl/fl^-WNT1 Cre embryos at E15 and E16.5, but there was no significant abnormity in the thickness of the ventricular wall and ventricular septum (Fig. 4F, G). More importantly, among Gltscr1^fl/fl^-WNT1 Cre, Gltscr1^fl/wt^-WNT1 Cre, and Gltscr1^fl/fl^ mice, the prognosis of Gltscr1^fl/fl^-WNT1 group was the worst in survival analysis over 150 days. Otherwise, the survival rate of Gltscr1^fl/fl^-WNT1 Cre mice at the 150th day was 20%, which was consistent with the percentage of heart defects at P0 (Fig. 2H).

To further confirm the Gltscr1^fl/fl^-WNT1 Cre mice presented heart defects which led to neonatal lethality. We measured cardiac function of Gltscr1^fl/fl^-WNT1 Cre and Gltscr1^fl/fl^ mice by ultrasonic cardiogram. It showed that the Gltscr1^fl/fl^-WNT1 Cre mice had a transventricular septal flow and a lower right ventricular output, which was consistent with the histological analysis (Fig. 4I–K). In addition, the cardiac function evaluation showed that Gltscr1^fl/fl^-WNT1 Cre mice had a lower ejection fraction (Figs. 4L, M, and S3B). Taken together, our data indicated that specific deletion of Gltscr1 in neural crest cells cause cardiac defects and neonatal lethality.

### Deletion of Gltscr1 leads to dysregulate of heart development-associated genes

**Fig. 5:**
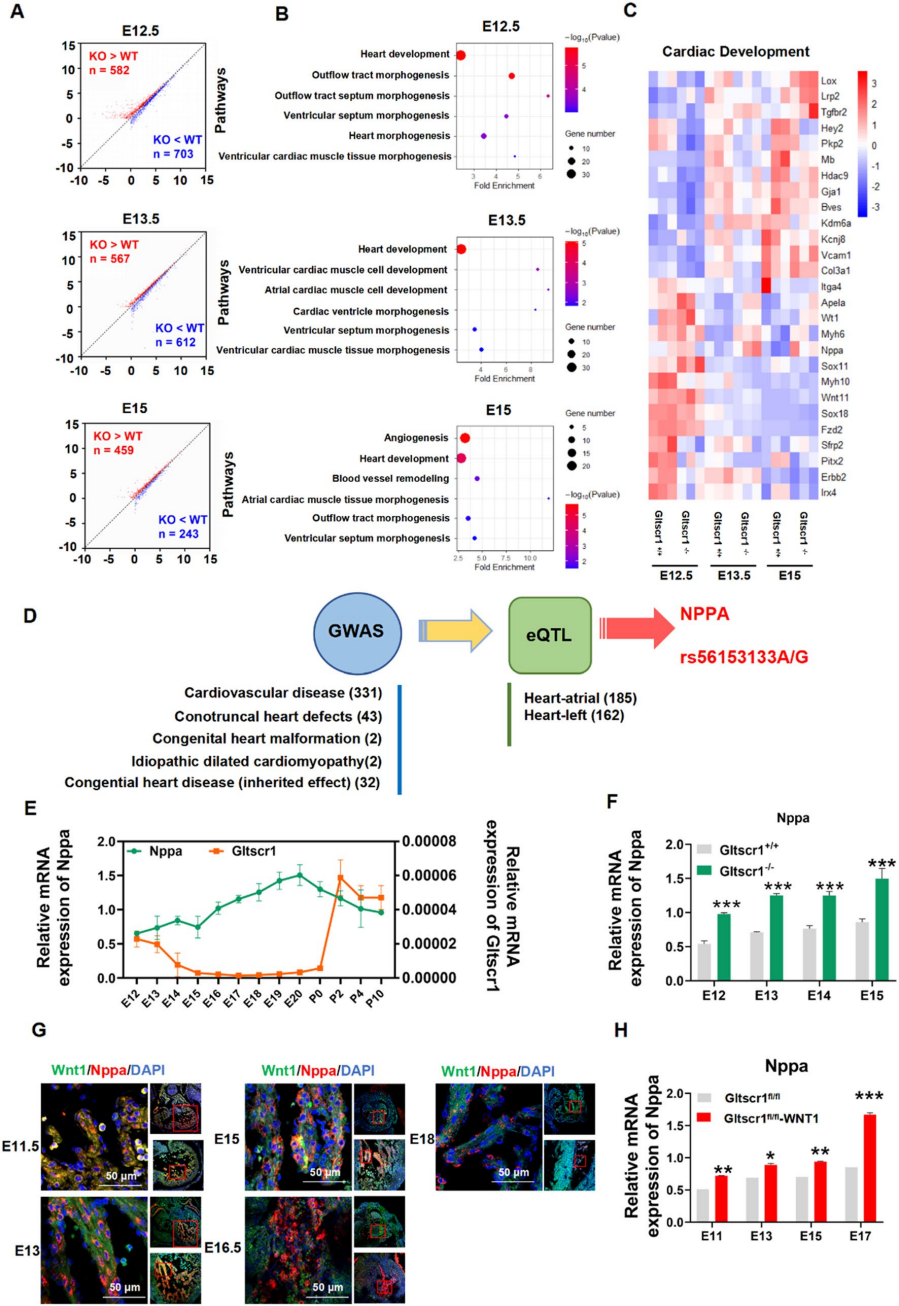
Gltscr1-deficient embryo dysregulates Nppa resulting in cardiac defects.** A** Scatter plot of the RNA-seq expression data to identify differentially expressed genes (DEGs) in embryonic heart tissues between Gltscr1^+/+^ and Gltscr1^−/−^ at the E12.5, E13.5, or E15 developmental stages. DEGs were defined as *P* < 0.05, fold change > 1.5, or fold change < 0.67. Red and blue dots represent up- and downregulated genes, respectively, in the Gltscr1^−/−^ group when compared to Gltscr1^+/+^. **B** Gene Ontology enrichment analysis was performed to analyze DEGs identified between Gltscr1^+/+^ and Gltscr1^−/−^ at the E12.5, E13.5, or E15 developmental stages. **C** Heatmap presentation of the cardiac development-associated DEGs in Gltscr1^+/+^ and Gltscr1^−/−^ heart tissue collected at E12.5, E13.5, or E15 developmental stages. **D** Screening strategy of congenital heart malformation-associated SNPs. **E** RT–qPCR detected the expression of Nppa and Gltscr1 in wild-type mouse heart tissues collected at different developmental stages (E12 to P10). **F** RT–qPCR detected the expression of Nppa in Gltscr1^+/+^ and Gltscr1^−/−^ heart tissues collected at different developmental stages (E12 to E15). **G** Immunofluorescence assay detected the colocalization with WNT1 and Nppa in heart tissue collected at E11.5 to E18 developmental stages, WNT1 in green, Nppa in red, and nucleus stain by DAPI in blue. Scale bar: 50 μm. **H** RT–qPCR detected the expression of Nppa in Gltscr1^fl/fl^ and Gltscr1^fl/fl^-WNT1 Cre embryonic heart tissues collected at E11 to E17 developmental stages. *P < 0.05, **P < 0.01, and ***P < 0.001 compared to control (two-tailed Student’s *t* test were used)

Previous studies identified GLTSCR1 as a transcription elongation factor and a member of the mammalian BAF complexes to regulate gene expression and genome integrity [4, 6]. To determine whether the congenital heart defects in Gltscr1 deletion mice were associated with the dysregulation of gene expression, we performed whole-genome transcriptional analysis (RNA-seq) in Gltscr1^−/−^ and Gltscr1^+/+^ cardiac tissues collected from E12.5, E13.5, and E15 embryos. Compared with Gltscr1^+/+^ mice, the cardiac tissues from Gltscr1^−/−^ mice carried many differentially expressed genes (DEGs) (Fig. 5A). Gene ontology (GO) enrichment analysis showed that the DEGs were enriched in several important heart development-associated biological processes, including heart development, ventricular cardiac muscle tissue morphogenesis, and atrioventricular valve development (Fig. 5B). Furthermore, the key genes involved in cardiovascular development, including Nppa, WT1, and Irx4, were significantly dysregulated in the heart development process of Gltscr1^−/−^ mice (Figs. 5C and S4A). Since Gltscr1 was reported expressed in both neurons and glia of adult brain in Drosophila, which is responsible for the male courtship learning and choice performance [27]. Therefore, we also performed RNA-seq in Gltscr1^−/−^ and Gltscr1^+/+^ brain tissues collected from E15 embryos and analyzed by GO enrichment analysis. Unfortunately, there was no nervous system development-associated pathway enriched (Fig. S4B, C).

Then, we collected data from five genome-wide association studies (GWASs) for cardiovascular disease (GCST007072) [28], conotruncal heart defects (GCST002438) [29], congenital heart malformation (GCST002036) [30], idiopathic dilated cardiomyopathy (GCST001023) [31], and congenital heart disease (inherited effect) (GCST004718) [32] from the NHGRI-EBI GWAS Catalog to obtain the SNPs associated with congenital heart malformations (June 2020) [33]. Interestingly, the expression quantitative trait loci (eQTL) analysis from the GTEx Project [34] showed that congenital heart malformation risk-associated SNP rs56153133 was associated with the expression of NPPA in atrial appendage tissue (*P* = 1.86E − 05) (Fig. 5D). As a key gene in heart development, Nppa is finely regulated with a highly cardiac restriction and dynamical expression pattern during mouse embryonic development [17]. However, Nppa expression was upregulated by Gltscr1 deletion. Therefore, we tried to gain further insight into the mechanism by which GLTSCR1 regulates NPPA in heart development. Therefore, we collected embryos of Gltscr1^+/+^ mice at different embryonic and postnatal time points from E12-P10. As shown in Fig. 5E, Nppa continually increased during embryonic progression but dropped dramatically at P0, which was consistent with previous studies. Interestingly, Gltscr1 expression gradually decreased from E12 to P0 but sharply increased after P0, which presented a negative correlation with Nppa from E12-P10. Furthermore, we detected the expression of Nppa in the embryos of Gltscr1^−/−^ and Gltscr1^+/+^ mice from E12 to E15 to verify the negative regulatory effect of Gltscr1 on Nppa expression (Figs. 5F and S4D). Consistently, the immunofluorescence assay showed that Nppa was colocalized in embryonic heart with WNT1 and cTNT (Figs. 5G and S4E), and conditional deletion of Gltscr1 in neural crest cells increased Nppa expression in heart at different cardiac development periods compared with control (Fig. 5H). Collectively, Gltscr1 deletion caused the cardiac defects though dysregulating heart development-associated genes.

### GLTSCR1 deletion coordinates the G allele of rs56153133 to increase the expression of NPPA

**Fig. 6:**
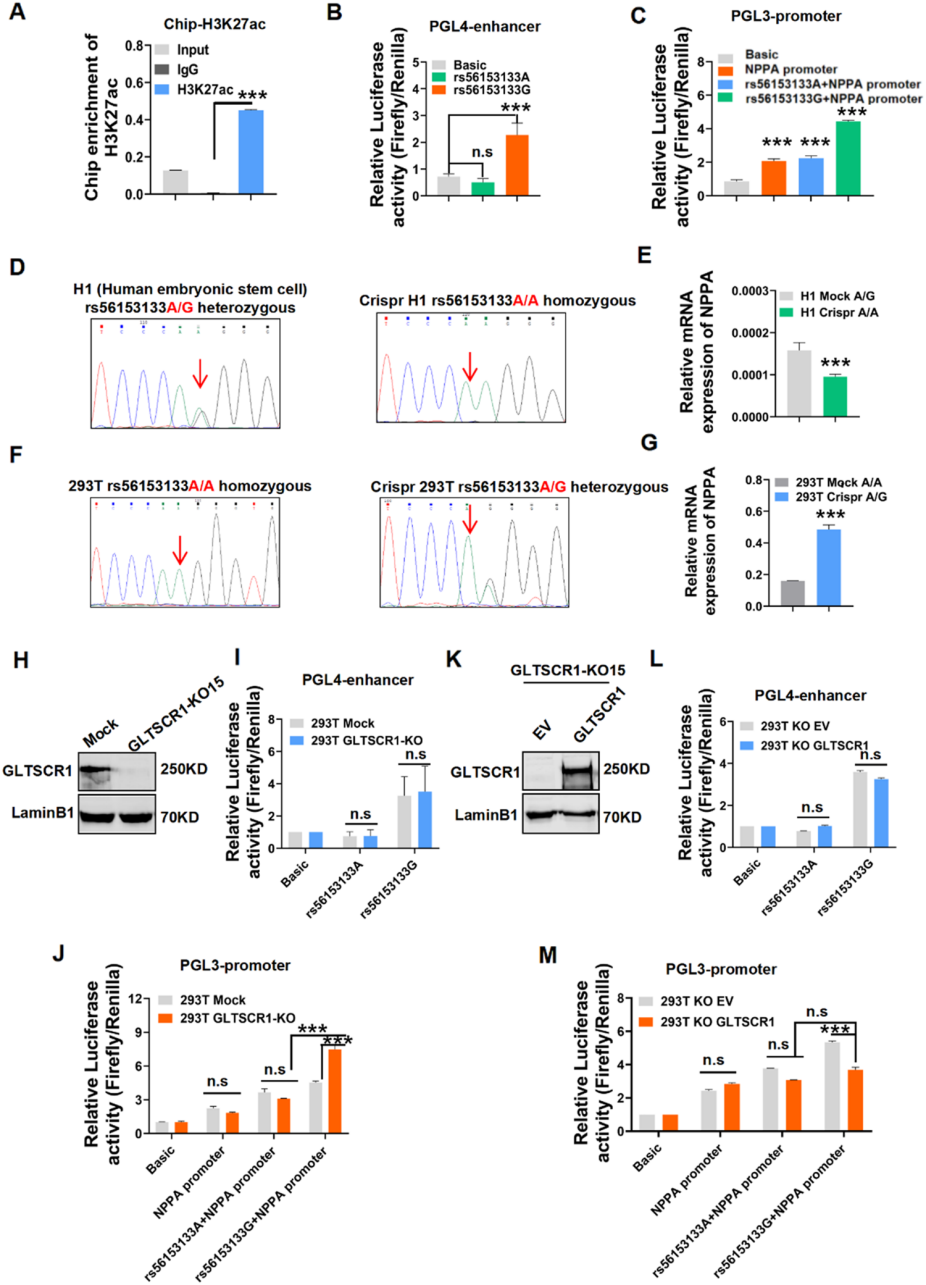
Gltscr1 deletion coordinates the G allele of rs56153133 to increase the expression of NPPA. **A** Chip-qPCR analysis of H3K27ac binding at the rs56153133 locus in HEK293T cells. **B**, **C** Luciferase reporter assays using vectors containing the SNP locus for either enhancer (chromosome 1:11824590–11825590) (**B**) or promoter (chromosome 1:11847682–11848865) assays (**C**) in HEK293T cells. The pGL4-promoter vector was used for the enhancer assay, and the pGL3-basic vector without a promoter was used for the promoter assay. Luciferase signals were normalized to Renilla signals (*n* = 3). **D**, **E** Sanger sequencing results of Crispr-edited H1 cells and wild-type H1 cells (**D**). RT–qPCR detected the expression of NPPA in both cell lines (**E**). **F**, **G** Sanger sequencing results of Crispr-edited HEK293 cells and wild-type HEK293 cells (**F**). RT–qPCR detected the expression of NPPA in both cell lines (**G**). **H** Western blotting detected GLTSCR1 knockout in HEK293 cells. **I**, **J** Luciferase reporter assays using vectors containing SNP loci for either enhancer (**I**) or promoter (**J**) assays to detect transcriptional activity in GLTSCR1-KO and mock cells. Luciferase signals were normalized to Renilla signals (*n* = 3). **K–M** Western blotting detected the re-expression of GLTSCR1 in GLTSCR1-KO HEK293 cells (**K**). Luciferase reporter assays using vectors containing SNP loci for either enhancer (**L**) or promoter (**M**) assays to detect transcriptional activity in GLTSCR1-KO cells transfected with empty vector and re-expressing GLTSCR1. Luciferase signals were normalized to Renilla signals (*n* = 3). *P < 0.05, **P < 0.01, and ***P < 0.001 compared to control (two-tailed Student’s t test were used)

As a key gene in heart development, Nppa expression was regulated by Gltscr1 and SNP rs56153133. To assess the regulatory potential of SNP rs56153133 to NPPA expression, we used a chromatin immunoprecipitation (ChIP) assay to detect the enrichment level of H3K27ac and the key histone modification of promoter and enhancer [35], for DNA fragment carrying SNP rs56153133. The result showed that the SNP rs56153133 region presented a high H3K27ac binding signal (Fig. 6A). Because of the distance of rs56153133 to the TSS, the region of SNP rs56153133 might be considered as an enhancer for regulating NPPA expression (Fig. S5A). The enhancer luciferase-based reporter assay showed that the G allele of rs56153133 significantly increased the activity of luciferase, but the A allele of rs56153133 did not (Fig. 6B). We also performed a luciferase-based promoter reporter assay by cloning the DNA fragment together with the NPPA promoter sequence into a luciferase reporter vector (Fig. S5A). As expected, the G allele of rs56153133 still increased the luciferase activity of the NPPA promoter, but the A allele of rs56153133 did not present such regulatory activity (Fig. 6C). To further confirm the role of SNP rs56153133 in regulating NPPA expression, we mutated the G allele of human embryonic stem cell H1 to the A allele at SNP rs56153133 (Fig. 6D). Interestingly, the mRNA expression of NPPA was significantly decreased in H1 cells with the AA genotype of rs56153133 (Fig. 6E). Furthermore, when we mutated the homozygous AA genotype of 293 T cells to the heterozygous AG genotype, the mRNA expression of NPPA was significantly increased (Fig. 6F, G). Otherwise, NPPB, the downstream gene of NPPA, also presented a similar expression change as NPPA, but no significant expression change in CLCN6 was observed in these mutant cells (Fig. S5B, C).

To determine the correlation of GLTSCR1 with the NPPA SNP rs56153133 for regulating NPPA expression, we knocked out GLTSCR1 (GLTSCR1-KO) in 293 T cells (Fig. 6H). The luciferase-based enhancer and promoter assays showed that deletion of GLTSCR1 did not influence the activity of the NPPA enhancer with the G or A allele of rs56153133 (Fig. 6I). The loss of GLTSCR1 exhibited a synergistic effect in increasing the activity of the NPPA promoter with the enhancer of the rs56153133 G allele (Fig. 6J). Consistently, the overexpression of GLTSCR1 in GLTSCR1-KO cells showed the same result (Fig. 6K–M). These results suggested that the loss of GLTSCR1 promoted NPPA expression by cooperating with the G allele of the rs56153133 enhancer activity.

### GLTSCR1 inhibits NPPA expression by blocking the interaction of ZNF740 with NPPA promoter

**Fig. 7:**
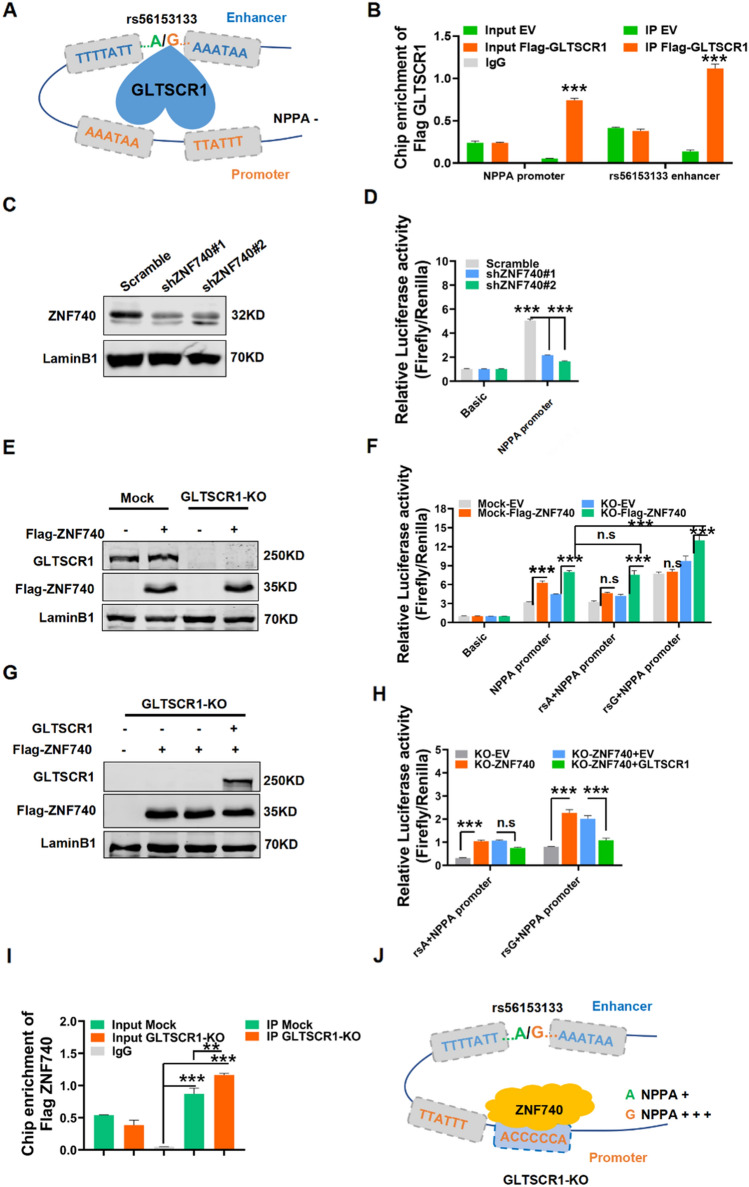
GLTSCR1 inhibits NPPA expression by blocking the interaction of ZNF740 in NPPA promoter.** A** The regulation model of GLTSCR1 to NPPA through binding NPPA enhancer and promoter. **B** Chip-qPCR analysis of GLTSCR1 binding at the NPPA promoter and the rs56153133 enhancer locus in HEK293T cells by anti-Flag beads. **C** Western blotting detected ZNF740 knockdown by shRNA in HEK293 cells. **D** Luciferase reporter assays using vectors containing the NPPA promoter to detect the transcriptional activity in ZNF740 knockdown cells. Luciferase signals were normalized to Renilla signals (*n* = 3). **E** Western blotting detected the overexpression of ZNF740 in HEK293 mock or GLTSCR1-KO cells. **F** Luciferase reporter assays using vectors containing the NPPA promoter or NPPA promoter with SNP locus enhancers to detect transcriptional activity in ZNF740-overexpressing mock or GLTSCR1-KO cells. Luciferase signals were normalized to Renilla signals (*n* = 3). **G** Western blotting detected ZNF740 overexpression and GLTSCR1 re-expression in GLTSCR1-KO HEK293 cells. **H** Luciferase reporter assays using vectors containing the NPPA promoter with the A or G allele of the rs56153133 SNP locus to detect the transcriptional activity of ZNF740 overexpression and GLTSCR1 re-expression in GLTSCR1-KO HEK293 cells. Luciferase signals were normalized to Renilla signals (*n* = 3). **I** Chip-qPCR analysis of ZNF740 binding at the NPPA promoter in mock and GLTSCR1-KO cells by anti-Flag beads. **J** The regulation model of ZNF740 to NPPA through binding NPPA promoter when GLTSCR1 is knockout. *P < 0.05, **P < 0.01, ***P < 0.001 compared to control (two-tailed Student’s t test were used)

To further investigate the molecular mechanism by which GLTSCR1 regulates NPPA expression, we analyzed the sequence of the NPPA enhancer carrying SNP rs56153133 and the NPPA promoter (− 1084 to   + 100 bp to TSS). As shown in Fig. 7A, there were two GLTSCR1-binding motifs in the NPPA enhancer spanning the SNP rs56153133 and two contiguous GLTSCR1-binding motifs in the NPPA promoter. Basing to these findings, we hypothesized that GLTSCR1 blocks some transcriptional activators of the NPPA promoter or enhancer by binding to these motifs. The ChIP assay showed that GLTSCR1 could bind to both the enhancer and promoter of NPPA (Figs. 7B and S6A). To further determine whether GLTSCR1 directly binds to these specific motifs in NPPA enhancer and promoter, we synthetized the biotin-labeled DNA probes containing the shared binding motif sequence of NPPA enhancer and promoter. Combining with immunoprecipitation-purified GLTSCR1 protein, the electrophoretic mobility shift assay showed that GLTSCR1 could block wild probe mobility shift, but not for the mutated probe (Fig. S6B), which demonstrated that GLTSCR1 could directly bind to the specific motif of NPPA enhancer and promoter. Furthermore, we used the online database Jaspar (https://jaspar.genereg.net/) to predict the transcription factor that bind to the NPPA enhancer or promoter region. Intriguingly, there was a ZNF740-binding site in the NPPA promoter that was located 53-bp upstream of the GLTSCR1-binding motif (Fig. S7C, D). Previously, ZNF740 was reported as a transcription factor of MEF2C, which effects the differentiation of stem cells into trophoblast [36]. Then, the luciferase-based promoter assay suggested that ZNF740 knockdown decreased the activity of the NPPA promoter (Fig. 7C). Then, overexpressed ZNF740 in GLTSCR1-KO and control (mock) cells significantly increased the luciferase activity of the NPPA promoter in both GLTSCR1-KO and mock cells. However, GLTSCR1-KO increased the promotion of rs56153133G on the NPPA promoter in the ZNF740-overexpressing group (Fig. 7E, F). Next, we overexpressed GLTSCR1 in GLTSCR1-KO cells along with ZNF740 overexpression. Interestingly, we found that the transcriptional activity of rs56153133G in the NPPA promoter was directly inhibited by rescuing GLTSCR1 overexpression, but there was no effect on the rs56153133A allele (Fig. 7G, H). To determine the competition binding between GLTSCR1 and ZNF740 in NPPA promoter, Chip assay was performed in GLTSCR1-KO and mock cells, and we observed GLTSCR1-KO had more ZNF740 enrichment in NPPA promoter-binding site (Fig. 7I). These data suggested that GLTSCR1 repressed NPPA expression by blocking the ZNF740-binding site in the NPPA promoter (Fig. 7J). Loss of GLTSCR1 released the binding site for ZNF740 to promote NPPA overexpression, which caused CHD.

## Discussion

Our previous studies demonstrated that GLTSCR1 inhibits colorectal cancer metastasis by modulating transcription elongation [4] and RNA alterative splicing [5]. Although it is required for transcription regulation and genome integrity [37, 38], the role of GLTSCR1 in heart development remains unclear. Here, we first demonstrated that GLTSCR1 was required for embryonic heart development. Gltscr1 deficiency resulted in embryonic lethality with cardiac defects. Otherwise, the Gltscr1 heterozygosity defect caused growth restriction in the early postnatal period.

By our knowledge, it is the first time to generate a Gltscr1 knockout mice strain to investigate its role in embryonic development and survival. Both two strains of Gltscr1 knockout mice exhibited VSD and DORV defects which led to embryonic lethality. Unexpectedly, conditional deletion of Gltscr1 in heart development such as myocardial and cardiovascular endothelial cells did not cause any cardiac defect and growth retardation. Different myocytic and nonmyocytic cell lineages of heart are generated from three precursor populations, including cardiogenic mesoderm cells (CMC), CNCCs, and the proepicardium (PE) [39]. The CNCCs is a subpopulation of the neural crest and a unique subregion of the cranial neural crest which migrates into the embryonic OFT and participates in development of cardiovascular system [40]. Abnormal development of the OFT and aortic arch (AA) account for ~ 30% CHD [41]. Conditional mutation N-cadherin and TGF-β receptor Alk2 in neural crest cells by WNT1 Cre leads to persistent truncus arteriosus (PTA) as the CNC cells fail to enter the OFT cushions [42, 43]. In addition, conditional loss of an ARID1A-containing SWI/SNF chromatin remodeling complexes in CNCCs results in embryonic lethality in mice with heart defects of incomplete cardiac CNCC colonization of the outflow tract and septation of the arterial trunk [44]. However, the role of GLTSCR1 as a member of SWI/SNF chromatin remodeling complex (GBAF) in heart development and CNCCs defects remains unclear. Therefore, we generated a specific deletion of Gltscr1 in neural crest cells. The Gltscr1 CKO mice caused a VSD and DORV cardiac defect, in which the cardiac phenotype also occurred in Gltscr1 TKO mice. Interestingly, Gltscr1 CKO in neural crest cells did not cause embryonic lethality, but led to neonatal lethality partly. Taken together, Gltscr1 CKO in neural crest cells partial phenotypic consistent with Gltscr1 TKO embryos. The more complex biological function of GLTSCR1 in embryonic development needs to be illustrated, including its regulation in growth hormone. These data demonstrated that GLTSCR1 played an important role in heart development, which dysregulation might be related to CHD.

As our previous study reported, a frameshift mutation in the sixth exon of GLTSCR1 results into losing function in colorectal cancer [4]. Allelic loss of the chromosome 19q arm with location of GLTSCR1 is a frequent event in human diffuse gliomas. Furthermore, the high methylation in cg01544805, cg16879871, and cg16128638 in promoter of GLTSCR1 also causes the downregulation of GLTSCR1. In addition, as a nuclear localization protein, the translocation of GLTSCR1 to cytoplasm causes loss of function in cancers. Due to limitations in the acquisition of clinical CHD samples, we could not provide more solid evidence for the role of GLTSCR1 in CHD. However, the variants or deletion of GLTSCR1 could be associated with CHD in embryonic development.

Embryonic heart development is a precise process that controlled by temporal and spatial regulation of cardiac transcriptional networks through receiving a multitude of intracellular and extracellular signals [45, 46]. NPPA is one of the best-characterized cardiac genes that regulating cardiac development and diseases. Along with embryonic development, NPPA expression is gradually increased, but decreased in postnatal period [18]. Our data not only confirmed NPPA expression pattern in heart development but also presented a unique dynamic cardiac expression pattern of GLTSCR1 that was gradually decreased, but increased in postnatal period. This negative relationship between GLTSCR1 and NPPA expression implied that GLTSCR1 might be considered as a NPPA transcription inhibitor. So far, some transcription factors to regulate NPPA have been reported, including Nkx2-5, GATA4, TBX5, and BAF60C. In the current study, ZNF740 was identified as a new transcription factor for NPPA. Intriguingly, GLTSCR1 could bind to both NPPA enhancer and promoter at some time that might not only change NPPA genome conformation but also block ZNF740 to interact with its promoter to repress NPPA transcription. Therefore, Gltscr1 deletion in neural crest cells caused an aberrant increase of NPPA, which led to cardiac defects and neonatal lethality. This interaction model of GLTSCR1 with enhancer/promoter to regulate NPPA transcription help us to further understand the molecular mechanism of NPPA dynamic expression in heart development. Although previous studies reported that GLTSCR1 encodes a subunit of the non-canonical BAF (GBAF) complex and regulates genes transcription [6, 7]. However, it needs further clarification whether our novel regulation model of GLTSCR1 for NPPA transcription was related to the function of GBAF complex. In addition, the embryonic lethality occurred in Gltscr1 TKO embryos at E16.5 might be a comprehensive regulation of GLTSCR1 in signaling network. Target genes of GLTSCR1 including but not just NPPA.

So far, several SNPs in NPPA genome have been identified to be associated with cardiovascular disease. SNP rs5063 was related to serum ANP levels and associated with hypertension in the Chinese Han population [33], and SNP rs17367504 was associated with blood pressure traits [16]. While, we found a new SNP rs56153133 in the NPPA enhancer, and the risk allele G of SNP rs56153133 was correlated with congenital heart disease. In addition, the eQTL result showed that SNP rs56153133 was associated with the expression of NPPA. More interestingly, SNP rs56153133 locates in the GLTSCR1-binding region. Our data demonstrated that the G allele of rs56153133 could significantly increase the expression of NPPA that caused by GLTSCR1 deficiency, but the A allele of rs56153133 had no such enhancer activity. Therefore, the increased risk of CHD by the G allele of rs56163133 could be attributable to the orchestrated effects of GLTSCR1 deficiency and its cooperation with the G allele of rs56153133. Otherwise, there are NPPB and a few other genes near the topologically associating domain (TAD) that has an insulate chromatin structure for NPPA localization. While, GLTSCR1 might just bind to TAD to create a local gene regulatory environment, which not only controlled NPPA but also NPPB expression. In conclusion, our study highlights the molecular mechanism of GLTSCR1 coordinating with SNP rs56153133 to regulate NPPA, which might become a potential biomarker and therapeutic target for CHD. But it needs further investigation for clinical application.

## Methods

This research complies with all relevant ethical regulations. Animal experiments performed in accordance with a protocol approved by the Institutional Animal Care and Use Committee at the Zhejiang University (Ethics Committee number: 17084).

### Mice

GLTSCR1^fl/wt^ and Gltscr1^+/−^ mice were generated by GemPharmatech Company (details of genomic editing by CRISPR show in support materials). To delete GLTSCR1 specifically in mouse myocardial cells, GLTSCR1^fl/fl^ mice was mated with cTNT-Cre mice (provided by Professor Ke Y, Zhejiang University, School of medicine) to generate GLTSCR1^fl/fl^-cTNT-Cre mice. To delete GLTSCR1 specifically in mouse cardiovascular endothelial cells, GLTSCR1^fl/fl^ mice was mated with CDH5 Cre mice (provided by Professor Ke Y, Zhejiang University, school of medicine) to generate GLTSCR1^fl/fl^-CDH5 Cre mice. To delete GLTSCR1 specifically in mouse neural crest cells, GLTSCR1^fl/fl^ mice was mated with WNT1 Cre mice (provided by Professor Maoqing Ye, Shanghai Key Laboratory of Clinical Geriatric Medicine, Huadong Hospital Affiliated to Fudan University) to generate GLTSCR1^fl/fl^-WNT1 Cre mice and GLTSCR1^fl/fl^ was used as controls. Genotypes were determined by PCR using genomic DNA.

### Cell culture and treatment

Human HEK293 cell line was purchased from the cell bank at the Chinese Academy of Sciences (Shanghai, China) and was cultured in Dulbecco’s modified Eagle medium. Human embryonic stem cell H1 was provided by Professor Hongwei Ouyang, Zhejiang University, School of medicine. H1 cells were cultured by mTeSR1 medium (Stem Cell). Cell lines were grown in a humidified atmosphere at 37 °C with 5% CO2. Plasmids were transfected into HEK293 cells with LipoD293 (SignaGen) and transfected into H1 cells with Lipofectamine™ stem reagent (STEM00001).

### Histological analysis

Paraffin-embedded mouse embryos and neonatal mouse hearts were cut into 5-μm sections and dewaxed in dimethylbenzene 10 min for three times, followed by rehydrating in a descending ethanol series (100%, 90%, 80%, 70%, and 50%). Then the sections were stained with hematoxylin and eosin for nuclei and cytoplasm stain. Finally, a coverslip was applied to the slide. Images were acquired on a NanoZoomer 2.0 HT analyzed by NDP software.

### Immunofluorescence assay

For immunohistochemical staining, rehydrated paraffin sections of mouse embryos were heated for 30 min in citrate antigen retrieval solution before blocking with 10% bovine serum albumin in TPBS (pH = 7.4); then, they were probed by primary antibodies (antibody details are provided in the supplementary Table 1) overnight at 4 °C, followed by incubation with fluorescent secondary antibodies for 1 h and another 20 min for DAPI stain. Then, sections were covered with coverslip by fluorescence quencher and acquired on Olympus IX83-FV3000-OSR.

### Chromatin immunoprecipitation assay

HEK293 cells were seeded in 10-cm culture dish. After 18 h, cells were transfected with Flag-GLTSCR1 by LipoD293. 48 h after transfection, cells were prepared for ChIP assay and used anti-Flag antibody for chromatin pull down. HEK 293 GLTSCR1-KO and Mock cells were seeded in 10-cm culture dish, after 18 h, cells were transfected with Flag-ZNF740, and 48 h after transfection, cells were prepared for ChIP assay and used anti-Flag antibody for chromatin pull down. SimpleChIP Enzymatic Chromatin IP Kit (Magnetic Beads) (Cell Signaling Technology, CAT No. 9003) was used for ChIP assays, which was performed according to the manufacturer’s instructions. Samples were analyzed by real-time PCR using SYBR Green Power Master Mix following the manufacturer’s protocol or by RT-PCR with agarose gel electrophoresis.

### Electrophoretic mobility shift assay

PCDNA3.1 + Flag-tagged GLTSCR1 vector and empty vector were overexpressed in 293 T, and proteins were enriched by M2 magnetic beads (Sigma, CAT#M8823). Purified proteins and biotin-labeled DNA (Motif probe F: TTTAAAATAAAAATT; Motif probe R: AATTTTTATTTTAAA; Mutated probe F: CCCGGGGCGGGGGCC; Mutated probe R: GGCCCCCGCCCCGGG) were incubated in Binding buffer by rotating at 37 °C for 4 h. Samples were diluted with loading buffer and loaded onto BeyoGel™ EMSA PAGE (Beyotime, CAT#GS302S). Electrophoresis was performed at 100 V for 1 h, followed by transfer to nylon membrane (Beyotime, CAT#FFN13) at 60 V for 1 h with a Bio-Rad transfer unit (Bio-Rad). After transfer, DNA was immobilized with UV cross-linker. The membrane was blocked with blocking buffer for 15 min at room temperature and then incubated with Streptavidin-HRP (LI 925-32230, LI-COR® BIOSCIENCES, USA) for 15 min. Membrane was washed three times with washing buffer and imaged by the Odyssey Infrared Imaging System (LI-COR Biosciences, Lincoln, NE, USA).

### RNA-seq

RNA of Gltscr1^−/−^ and Gltscr1^+/+^ cardiac tissues collected from E12.5, E13.5, and E15 embryos were extracted, sequenced, and analyzed by bioacme (Wuhan, China). Each group collected three embryos from the same parent and were used for condition. The cDNA libraries were prepared from high-quality RNA using an Illumina TruSeq RNA sample prep kit following the manufacturer’s instructions (Illumina, San Diego, CA, USA). The individual RNA-seq libraries were pooled based on their respective sample-specific 6-bp adaptors and sequenced at 150-bp/sequence pair-read using an Illumina NovaSeq system. Clean Reads were mapping into the hg19 reference genome by STAR and quantified by RSEM. Differential expression genes were identified by DESeq2 in R. Benjamini–Hochberg false discovery rate method was applied to correct for multiple hypothesis testing. Genes with *P* < 0.05, fold change > 1.5, or fold change < 0.67 were defined as different expression genes as candidates for further analysis. Gene expression Heatmap was accomplished with R package pheatmap. Gene Ontology enrichment analysis was performed using DAVID. The results were visualized by the R package ggplot2 in R software. All raw and processed sequencing data generated in this study have been submitted to the NCBI Sequence Read Archive (SRA, https://www.ncbi.nlm.nih.gov/sra) under accession number PRJNA820129.

### ELISA

Thyroid-stimulating hormone and growth hormone levels in serum at different growth time points were detected by ELISA kit (Reddo Biotech). According to the manufacturer’s instructions, first, prepare all reagents, samples, and standards. Add 50-μl standards or sample to each well, then add 50-μl Detection Reagent A and immediately shake and mix, and then incubate 1 h at 37 °C. Aspirate and wash three times, add 100 μl prepared Detection Reagent B, and then incubate 20 min at 37 °C. Aspirate and wash 5 times again. Add 90-μl Substrate Solution and incubate 20 min at 37 °C. Finally add 50-μl Stop Solution and read at 450 nm immediately.

### Ultrasound scan

To identify heart defects, 6-week mice were ultrasound scanned with a two-tier ultrasound phenotyping strategy using VEVO LAZR-X ultrasound systems. Images were acquired and analyzed by Vevo LAB 3.2.6.

### SNP and eQTL analyses

SNPs associated with congenital heart malformations were retrieved from the NHGRI-EBI GWAS Catalog (June 2020; https://www.ebi.ac.uk/gwas/home) [33]. Then, eQTL signals in heart tissues including atrial appendage (AA) and left ventricle (LV) were derived from the GTEx project (v8; https://www.gtexportal.org/home/) [34].

### Quantifications and statistical analysis

Statistical specifications of each experiment precision measures (mean and ± standard error of mean) and the statistical tests used are provided in the figures and figure legends. The following designations for levels of significance were used within this manuscript: *P < 0.05; **P < 0.01; ***P < 0.001; ns, not significant.

## Supplementary Information

Below is the link to the electronic supplementary material.

Supplementary file1 (DOCX 34721 kb)

Supplementary file2 (DOCX 15 kb)
