# Supplementary material for: Loss of GLTSCR1 causes congenital heart defects by regulating NPPA transcription
Source: Angiogenesis. 2023 Feb 6;26(2):217–32. doi: 10.1007/s10456-023-09869-6 (PMC10119265; doi:10.1007/s10456-023-09869-6)
Supplement: Supplementary file 1 — Supplementary file1 (DOCX 34721 kb) [file 10456_2023_9869_MOESM1_ESM.docx]

**Supplementary figures**

**Loss of GLTSCR1 causes congenital heart defects by regulating NPPA transcription**

Fengyan Han^1, 2, 3, #^, Beibei Yang^1, #^, Yan Chen^1^, Lu Liu^1^, Xiaoqing Cheng^4^, Jiaqi Huang^4^, Ke Zhou^5^, Dandan Zhang^6^, Enping Xu^1,7, 8^, Maode Lai^7, 8, 9^, Bingjian Lv^10^, Hongqiang Cheng^4, *^, Honghe Zhang^1, 7, 8,^ ^*^

**Affiliations**

1 Department of Pathology and Women's Hospital, Zhejiang University School of Medicine, Research Unit of Intelligence Classification of Tumor Pathology and Precision Therapy, Chinese Academy of Medical Sciences (2019RU042), Hangzhou 310058, Zhejiang, China.

2 Key Laboratory of Aging and Cancer Biology of Zhejiang Province, Hangzhou, China.

3 Department of Pathology and Pathophysiology, School of Basic Medical Sciences, Hangzhou Normal University, Hangzhou, China

4 Department of Pathology and Pathophysiology and Department of Cardiology at Sir Run Run Shaw Hospital, Zhejiang University School of Medicine, Hangzhou 310058, China.

5 Center for Stem Cells and Regenerative Medicine, Department of Orthopedic Surgery of the Second Affiliated Hospital, Zhejiang University School of Medicine, Hangzhou 310058, China

6 Department of Pathology, and Department of Medical Oncology of the Second Affiliated Hospital，Zhejiang University School of Medicine, Hangzhou 310058, Zhejiang, China

7 Cancer Center, Zhejiang University, Hangzhou 310058, Zhejiang, China

8 Key Laboratory of Disease Proteomics of Zhejiang Province, Hangzhou 310058, Zhejiang, China.

9 Department of Pharmacology, China Pharmaceutical University, Nanjing, 210009, China.

10 Department of Pathology and Women's Hospital, Zhejiang University School of Medicine, Hangzhou 310058, Zhejiang, China.

# These authors contribute equally.

*Correspondence: Prof. Honghe Zhang, Department of Pathology and Women's Hospital, Zhejiang University School of Medicine, Hangzhou 310058, China; honghezhang@zju.edu.cn. Dr Hongqiang Cheng, Department of Pathology and Pathophysiology and Department of Cardiology at Sir Run Run Shaw Hospital, Zhejiang University School of Medicine, Hangzhou, China. Hqcheng11@zju.edu.cn.


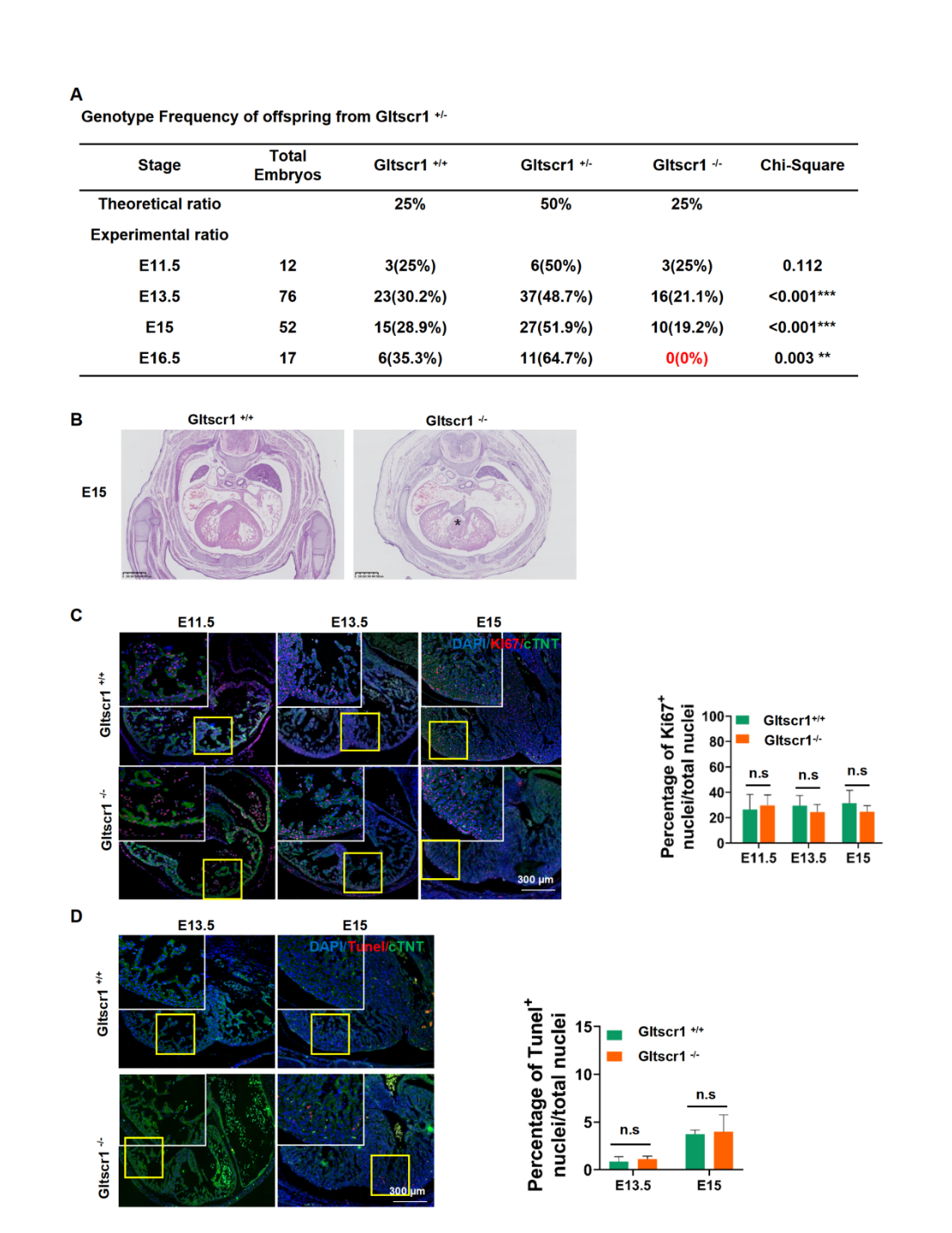


**Fig. S1** Gltscr1 deficiency causes cardiac development defects. **A** Genotyping analysis from Gltscr1^+/+^ and Gltscr1^+/-^ interbreeding. **B** Representative H&E staining images of Gltscr1^+/+^ and Gltscr1^+/-^ embryonic heart tissues collected at E15, the arrow indicates the DORV. Scale bar: 500 µm. **C-D** Immunofluorescence assay detected ki67 (**C**) for cell proliferation and Terminal dUTP Nicked-End Labelling (TUNEL) assay (**D**) to detect apoptosis in heart tissue collected at E11.5, E13.5 and E15 developmental stages, cTNT in green, ki67 or Tunel in red and nucleus stain by DAPI in blue. The right histogram are the qualification of **C** and **D**. The scale bar stands 300 μm.


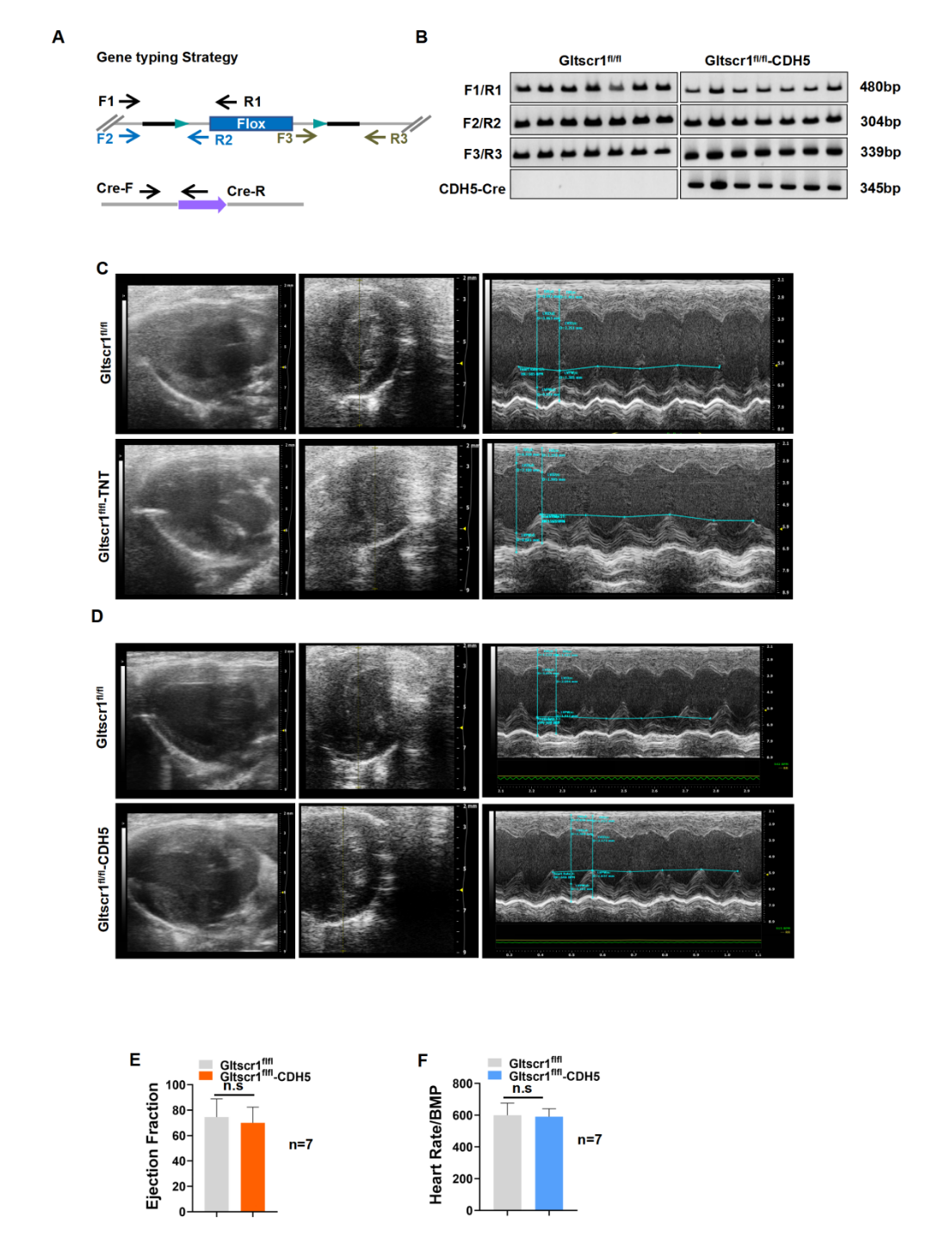


**Fig. S2** Conditional deletion of Gltscr1 in myocardial cells and cardiovascular endothelial cells. **A** Genotyping model of the Cre-loxp cyclization recombination enzyme system. **B** Genotype of conditional deletion Gltscr1 in cardiovascular endothelial cells. **C** Ultrasonic cardiogram of Gltscr1^fl/fl^ and Gltscr1^fl/fl^-TNT Cre mice. **D** Ultrasonic cardiogram of Gltscr1^fl/fl^ and Gltscr1^fl/fl^-CDH5 Cre mice. **E-F** The ejection fraction (**E**) and heart rate (**F**) in cardiovascular endothelial cells conditional Gltscr1 deletion mouse lines, n=7 (two-tailed Student’s t test was used).


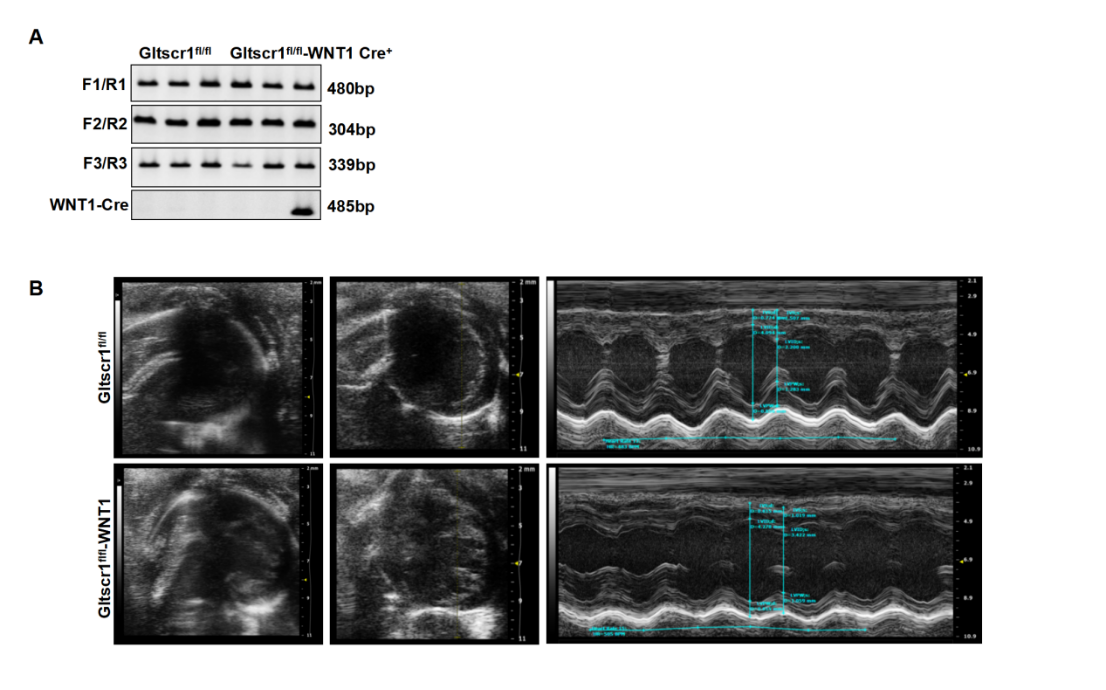


**Fig. S3** Specific deletion of Gltscr1 in neural crest cells. **A** Genotyping of the Gltscr1^fl/fl^-WNT1 Cre mouse strain. **B** Ultrasonic cardiogram of Gltscr1^fl/fl^ and Gltscr1^fl/fl^-WNT1 Cre mice.


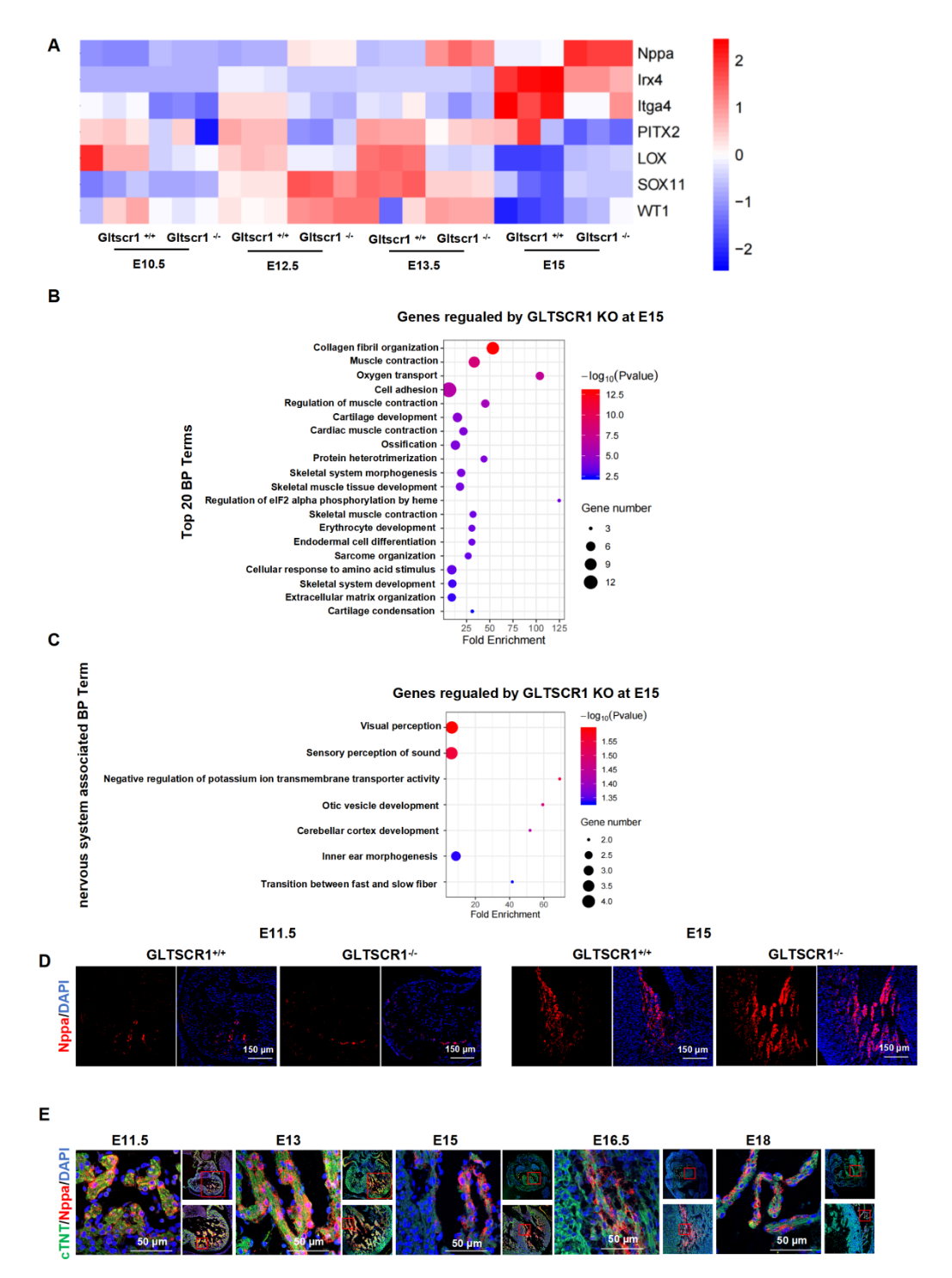


**Fig. S4** Gltscr1-deficient embryo exhibits cardiac defects with dysregulation of heart development-associated genes. **A** Verification of RNA-seq results in the cardiac development-associated DEGs in Gltscr1^+/+^ and Gltscr1^-/-^ heart tissue collected at E10.5, E12.5, E13.5 or E15 developmental stages by RT–qPCR. **B-C** Gene Ontology enrichment analysis was performed to analyze DEGs identified between Gltscr1^+/+^ and Gltscr1^-/-^ brains at the E15 developmental stage. **D** Immunofluorescence assay detected the Nppa in heart tissue collected at E11.5 and E15 developmental stages of GLTSCR1^+/+^ and GLTSCR1^-/-^ ; Nppa is shown in red, and DAPI is shown in blue. Scale bar: 150 μm. **E** Immunofluorescence assay detected the colocalization of cTNT and Nppa in heart tissue collected at E11.5 to E18 developmental stages of GLTSCR1^+/+^; cTNT is shown in green, Nppa is shown in red, and DAPI is shown in blue. Scale bar: 50 μm.


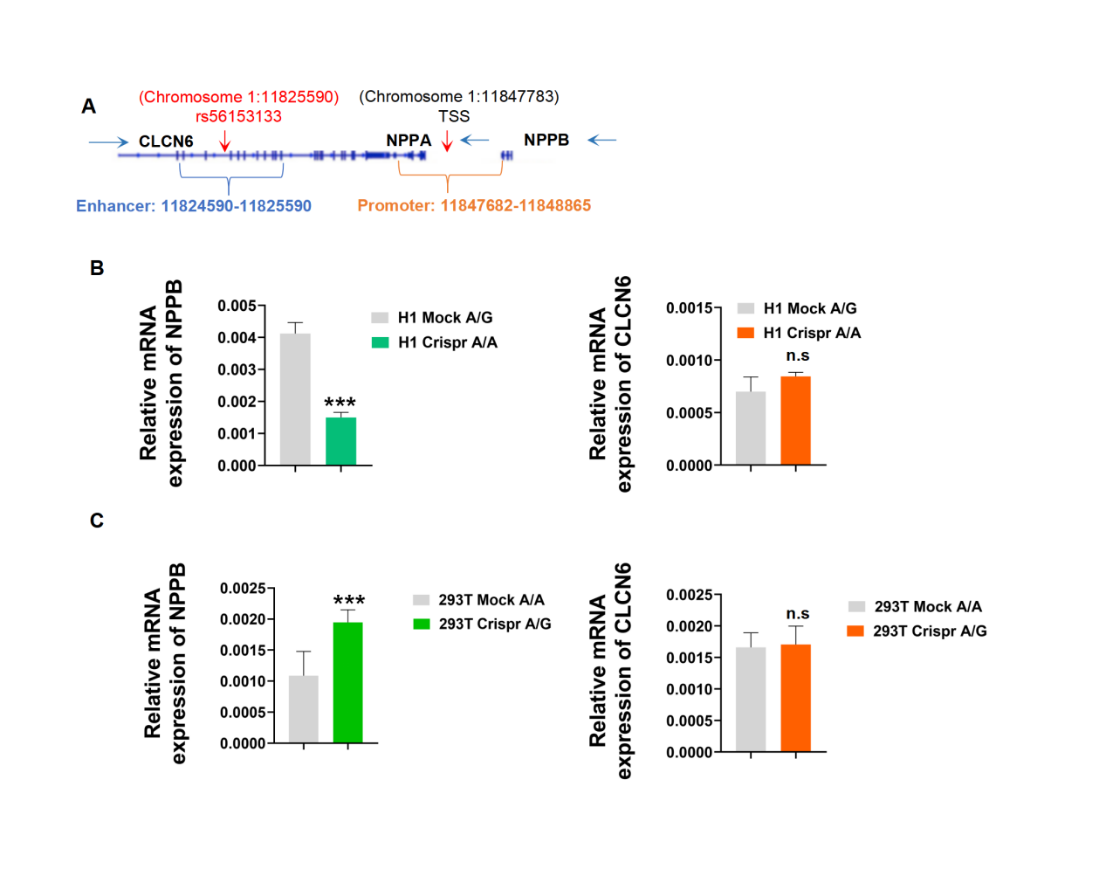


**Fig. S5** Gltscr1 deletion coordinates the G allele of rs56153133 to increase the expression of NPPA. **A** Schematic overview of SNP rs56153133 and the detail of enhancer and promoter location of NPPA in chromatin. The arrow in blue stands for the direction of gene transcription. **B-C** RT–qPCR detected the expression of NPPB and CLCN6 in CRISPR-edited H1 (**B**) or HEK293 (**C**) cells.

**
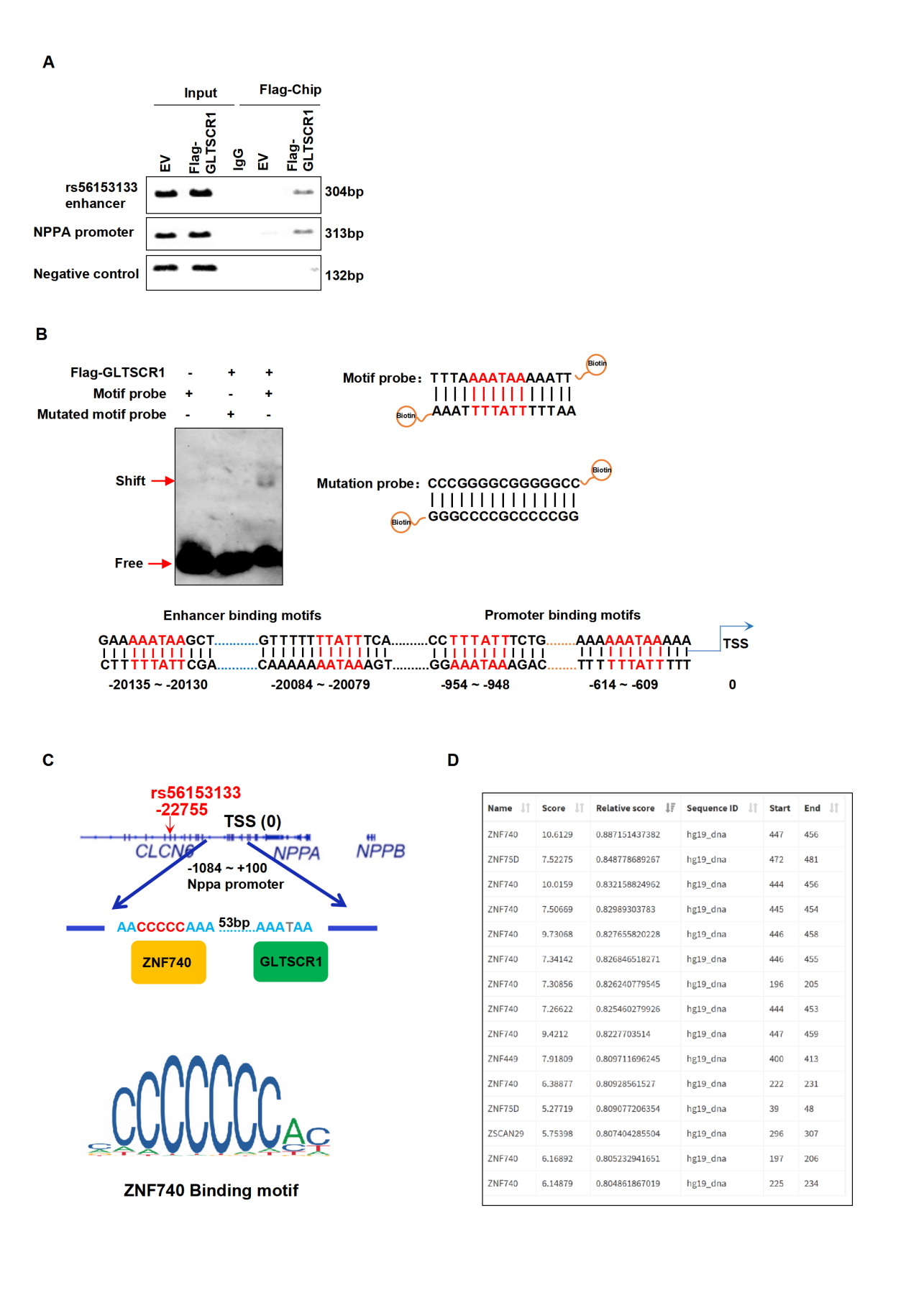
**

**Fig. S6** GLTSCR1 inhibits NPPA expression by blocking the interaction of ZNF740 with the NPPA promoter. **A** Chip-PCR [gel](javascript:;) [electrophoresis](javascript:;) analysis of GLTSCR1 binding at the NPPA promoter and the rs56153133 enhancer locus in HEK293T cells by anti-Flag beads. **B** electrophoretic mobility shift assay detected binding capacity of GLTSCR1 to its motif in vitro. **C** Schematic overview of rs56153133 and the NPPA promoter location in chromatin. The picture shows the GLTSCR1 and ZNF740 binding motifs in the NPPA promoter. **D** The predicted binding motif of ZNF740 in NPPA promoter.
