# Supplementary material for: Loss of GLTSCR1 causes congenital heart defects by regulating NPPA transcription
Source: Angiogenesis. 2023 Feb 6;26(2):217–32. doi: 10.1007/s10456-023-09869-6 (PMC10119265; doi:10.1007/s10456-023-09869-6)
Supplement: Supplementary file 2 — Supplementary file2 (DOCX 15 kb) [file 10456_2023_9869_MOESM2_ESM.docx]

**Supplementary table 1. The antibodies used in this study**

| Mouse monoclonal anti-Wnt1 (E-10) | Santa | sc-514531 |
| --- | --- | --- |
| Rabbit monoclonal anti-[Natriuretic peptides A antibody](https://www.abcam.cn/natriuretic-peptides-a-antibody-ab126149.html) | Abcam | Ab209232 |
| Rabbit monoclonal anti-Cardiac Troponin T | Abcam | Ab209813 |
| Monoclonal ANTI-FLAG® M2 antibody | Sigma | F1804-50UG |
| Mouse monoclonal anti-Ki67 | Abcam | Ab279653 |
| Rabbit monoclonal anti-LamiB1 | Abcam | ab133741 |
| Rabbit polyclonal anti-ZNF740 | Abcam | Ab122290 |
| Mouse monoclonal anti-GLTSCR1 | Produced by our lab |  |
| Alexa® Fluor 488 Donkey anti-Mouse IgG (H+L) | ThermoFisher | Cat#A-21202 |
| Alexa® Fluor 546 Goat anti-Rabbit IgG (H+L) | ThermoFisher | Cat#A-11010 |
| Alexa® Fluor 488 Goat anti-Rabbit IgG (H+L) | LI-COR | Cat#A-11008 |
| IRDye® 800CW Goat-anti-Rabbit Antibody | LI-COR | Cat#926-32211 |
| IRDye® 680CW Goat-anti-Mouse Antibody | LI-COR | Cat#926-68070 |
